# Efficacy and safety of radiotherapy combined with anti‐angiogenic therapy and immune checkpoint inhibitors in MSS/pMMR metastatic colorectal cancer

**DOI:** 10.1002/cam4.6820

**Published:** 2023-12-19

**Authors:** Menglan Zhai, Zixuan Zhang, Haihong Wang, Jinghua Ren, Sheng Zhang, Mingjie Li, Lichao Liu, Lisha Li, Lan Zhang, Xin Li, Tao Zhang, Zhenyu Lin

**Affiliations:** ^1^ Cancer Center, Union Hospital, Tongji Medical College Huazhong University of Science and Technology Wuhan China; ^2^ Queen Mary School, Medical Department Nanchang University Nanchang Jiangxi China; ^3^ Hubei Key Laboratory of Precision Radiation Oncology Wuhan China; ^4^ Department of Radiology Union Hospital, Tongji Medical College, Huazhong University of Science and Technology Wuhan China; ^5^ Institute of Radiation Oncology Union Hospital, Tongji Medical College, Huazhong University of Science and Technology Wuhan China

**Keywords:** immunotherapy, metastatic colorectal cancer, microsatellite stable, radiotherapy, targeted therapy

## Abstract

**Purpose:**

Several studies have demonstrated the effectiveness of anti‐angiogenic drugs in combination with immune checkpoint inhibitors (ICIs) in patients with microsatellite stable (MSS) or mismatch repair proficient (pMMR) metastatic colorectal cancer (mCRC). However, whether combination radiotherapy (RT) can further improve the prognosis of mCRC patients after second‐line treatment remains to be explored.

**Methods:**

Retrospective analysis of data from mCRC patients who received anti‐angiogenic targeted therapy (TT) and immunotherapy (IT) with or without RT after the failure of standard therapy. Progression‐free survival (PFS), overall survival (OS), objective response rate (ORR), disease control rate (DCR), and safety were evaluated.

**Results:**

A total of 82 patients who received TT + IT were analyzed. For RT group (*n* = 42) versus NRT group (*n* = 40), ORR was 21.4% (9/42) versus 5.0% (2/40); DCR was 83.8% (35/42) versus 65.0% (26/40). Compared with NRT group, RT improved PFS (median: 5.0 vs. 3.6 months; *p* = 0.04) and OS (median: 15.2 vs. 7.2 months; *p* = 0.01). In addition, in the population receiving RT, the PFS of RT sequential/simultaneous TT + IT was superior to TT + IT sequential RT (median: 7.1 vs. 6.2 vs. 3.5 months, *p* = 0.004). Multivariate analysis suggested RT was an independent prognostic factor for PFS and OS. No treatment‐related deaths were reported.

**Conclusions:**

Compared with TT + IT, RT combined with TT + IT improved survival outcomes in MSS/pMMR mCRC patients, with manageable toxicity. RT sequential/simultaneous TT + IT treatment is expected to be the optimal strategy for MSS/PMMR mCRC.

## 1 INTRODUCTION

With the rapid development of immunotherapy (IT), the era of IT for colorectal cancer (CRC) has begun, but the advantageous population is limited to less than 5% of high microsatellite instability (MSI‐H) patients, and 95% of microsatellite stable (MSS) or mismatch repair proficient (pMMR) type metastatic colorectal cancer (mCRC) is still difficult to benefit from immune checkpoint inhibitors (ICIs) therapy.^1^ The mechanisms of primary resistance to ICIs therapy in patients with MSS/pMMR‐type CRC are complex, which involve many factors in the tumor cells themselves as well as in the tumor microenvironment. Tumor neovascularization plays an important role in the tumor microenvironment. Previous studies have shown that vascular endothelial growth factor (VEGF) is involved in the regulation of tumor angiogenesis and immune microenvironment.^2^ Inhibition of tumor angiogenesis‐related pathways can not only inhibit neovascularization, but also improve the tumor microenvironment, with an immunomodulatory effect. In addition, anti‐angiogenic drugs and ICIs can work together on the tumor microenvironment, remodeling the tumor vascular microenvironment and immune microenvironment, converting the immune suppression state to the immune promotion state, increasing T cell infiltration to the tumor, and the combination of the two plays a synergistic anti‐tumor effect.^3^

Based on the above theoretical basis, in recent years, many attempts have been made in the combined application of anti‐angiogenic drugs and ICIs in the posterior line treatment of mCRC. Several small sample studies have shown preliminary efficacy, but the results are inconsistent (Table S1). RT is widely used in the treatment of malignant tumors, which can induce tumor immunogenic cell death, increase tumor immunostimulatory cell infiltration, enhance neoantigen expression, and transform immune “cold” tumors into immune “hot” tumors.^4^ The combination of RT, anti‐angiogenic agents, and ICIs has been reported to be a promising therapeutic modality in the field of hepatocellular carcinoma and glioma.^5^, ^6^, ^7^ Therefore, we speculate that RT in combination with anti‐angiogenic drugs and ICIs may further improve the efficacy of pMMR mCRC patients. In this study, we retrospectively analyzed the data of real‐world mCRC patients and found that adding RT to the course of the disease could significantly improve the survival benefit of anti‐angiogenic drugs combined with ICIs post‐line therapy in patients with MSS/pMMR mCRC.

## 2 PATIENTS AND METHODS

### 2.1 Patients

Retrospective analysis of patients with primary stage IV or recurrent metastatic CRC admitted to Cancer Center, Tongji Medical College, Union Hospital, Huazhong University of Science and Technology from January 2015 to September 2022. The main inclusion criteria for this study were as follows: (1) histopathologically confirmed CRC adenocarcinoma; (2) imaging diagnosis of primary stage IV or recurrent metastatic disease (including simultaneous metastasis: metastases occurring before or at the time of CRC diagnosis and within 6 months of radical surgery); (3) prior treatment with at least standard second‐line therapy (including irinotecan, oxaliplatin, and fluorouracil analogs) and treatment failure; (4) no prior treatment with PD‐1 monoclonal antibodies (mAb); (5) adequate organ function; (6) Eastern Cooperative Oncology Group performance status (ECOG PS) of 0–2; (7) and with at least a measurable lesion. Patients with other malignancy histories, and/or the presence of a serious comorbidity, such as heart, liver, lung, kidney, or blood system diseases, and patients with non‐anti‐angiogenic drugs combined with IT were excluded.

### 2.2 Treatment

In this study, PD‐1 inhibitors included sintilimab, nivolumab, camrelizumab, pembrolizumab, tislelizumab, and toripalimab. PD‐1 inhibitors were administered at the following dose: toripalimab 240 mg intravenous every 3 weeks; sintilimab, nivolumab, camrelizumab, pembrolizumab, and tislelizumab 200 mg intravenous every 3 weeks. The anti‐angiogenic drugs included fruquintinib and regorafenib. Patients received oral fruquintinib 3–5 mg or regorafenib 80–160 mg once a day for 21 consecutive days in 28–day cycles. Dose adjustment was allowed for treatment‐related toxicity. Treatment was continued until disease progression or intolerable toxicity. Patients underwent three‐dimensional conformal radiotherapy (3D‐CRT), intensity‐modulated radiation therapy (IMRT), and stereotactic body radiation therapy (SBRT). In general, 95% of the planning target volume at the prescribed dose is required, limiting the doses to the organ at risks (OARs). Details of RT were summarized in Table 2.

### 2.3 Tumor MMR/MSI status testing

Tumor MMR/MSI status was determined by examining either the loss of protein expression by immunohistochemistry of four MMR enzymes (MLH1/MSH2/MSH6/PMS2) or analysis of five tumor mononucleotide loci using polymerase chain reaction (PCR)‐based assays (five mononucleotide loci [BAT25, BAT26, NR21, NR24, and Mono27] or five mixed mononucleotide and dinucleotide loci [BAT25, BAT26, D17‐S250, D2S123, and D5S346]) using formalin‐fixed paraffin‐embedded tissue specimens. Tumors were defined as pMMR if all four MMR proteins were expressed in the tumor, and dMMR was defined when at least one MMR protein was absent. Specimens were classified as MSI‐H if at least two allelic loci of the five microsatellite markers analyzed were unstable, and as MSS if all five loci were stable. Tumors were classified as pMMR/MSS if either pMMR and/or MSS were found; tumors were classified as dMMR/MSi‐H if either dMMR and/or MSI‐H were found. Regarding the sample source for MMR/MSI status, 78 cases were based on primary focus and four cases on metastatic focus.

### 2.4 Assessment

The primary study endpoints were progression‐free survival (PFS) and overall survival (OS). The secondary endpoints were disease control rate (DCR), objective response rate (ORR), and the incidence of treatment‐related adverse events (TRAEs). Patients were evaluated by computed tomography every two or three systemic treatment cycles until disease progression or loss to follow‐up. Evaluation of tumor response according to the Response Evaluation Criteria in Solid Tumors (RECIST) version 1.1. The ORR was defined as the proportion of patients with the best overall response of complete response (CR) or partial response (PR). DCR was defined as the proportion of patients with the best overall response of CR, PR, or stable disease (SD). PFS was defined as the period from the date of initiation of TT + IT until disease progression, death from any cause, or the deadline for the last follow‐up (whichever occurred first). OS was from the beginning of TT + IT until the date of death as a result of any cause or the deadline of the last follow‐up. TRAEs were evaluated throughout the National Cancer Institute Common Terminology Criteria for Adverse Events (CTCAE), version 5.0.

### 2.5 Statistical analysis

PFS and OS were examined by the Kaplan–Meier method with a log‐rank test and the median follow‐up time was calculated using the inverse Kaplan–Meier method. Subgroup analysis of PFS and OS was carried out according to the baseline factors, and the results are presented as forest plots. Univariate analysis and multivariate Cox regression model were used to determine the influencing factors of PFS and OS. All statistical analyses were performed using SPSS version 25.0 software (IBM Corporation, Chicago, IL, USA). Continuous variables were expressed as median (range) and differences between groups were tested by *t*‐test, while categorical variables were represented by frequencies or percentages, and differences were analyzed by the Chi‐squared test or Fisher's exact test. The bilateral *p* < 0.05 was statistically significant.

## 3 RESULTS

### 3.1 Patient characteristics

**FIGURE 1:**
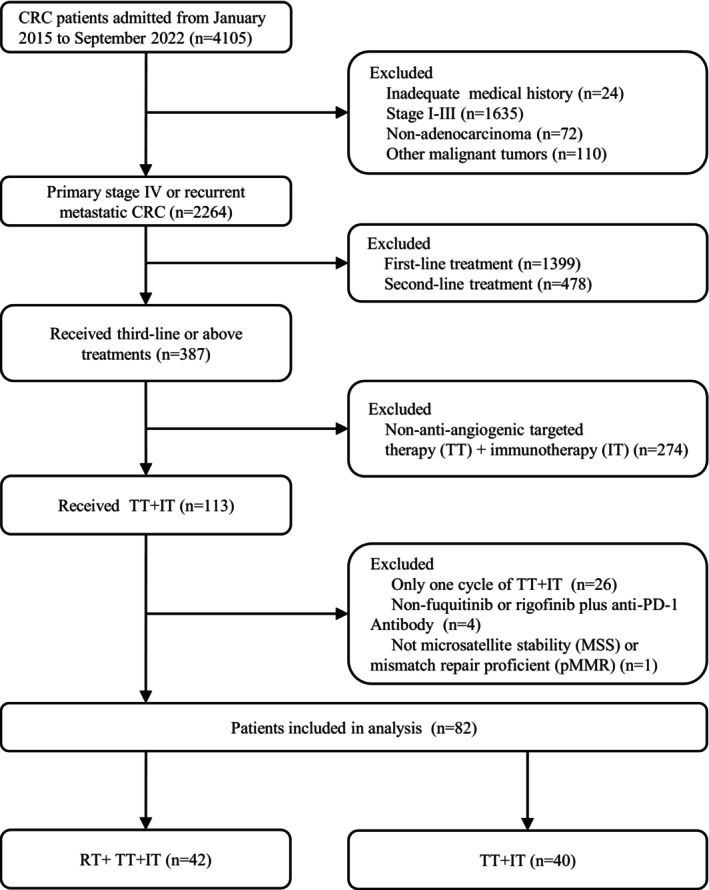
Patients screening flow chart.

We included 4105 patients who were diagnosed with CRC between January 2015 and September 2022 at the Cancer Center, Union Hospital, Tongji Medical College, Huazhong University of Science and Technology. Among them, 113 mCRC patients received targeted drugs plus ICIs. Finally, a total of 82 patients (42 in the RT group, and 40 in the NRT group) were included in the analysis (Figure 1). Baseline characteristics were balanced between the two groups (Table 1 **)**. The gender composition of the patients was similar in both groups.

**TABLE 1:** Baseline clinical characteristics of patients.

Characteristic	Total *n* (%)	RT group *n* (%)	NRT group *n* (%)	*p*‐value
Patients, *n* (%)	82	42 (51.2)	40 (48.8)	‐
Age, median (range)	56 (27–75)	58 (27–75)	55 (34–71)	0.57
Sex				0.65
Male	39 (47.6)	21 (50.0)	18 (45.0)	
Female	43 (52.4)	21 (50.0)	22 (55.0)	
ECOG PS				0.78
0	32 (39.0)	17 (40.5)	15 (37.5)	
1	50 (61.0)	25 (59.5)	25 (62.5)	
Cancer type				0.26
Primary metastatic disease	44 (53.7)	20 (47.6)	24 (60.0)	
Recurrent metastatic disease	38 (46.3)	22 (52.4)	16 (40.0)	
Primary tumor location				0.54
Rectum	32 (39.0)	18 (42.9)	14 (35.0)	
Left colon	24 (29.3)	13 (31.0)	11 (27.5)	
Right colon	26 (31.7)	11 (26.2)	15 (37.5)	
Metastases location				
Liver	50 (61.0)	27 (64.3)	23 (57.5)	0.53
Lung	50 (61.0)	30 (71.4)	20 (50.0)	0.05
Peritoneum	15 (18.3)	7 (16.7)	8 (20.0)	0.70
Lymph node	38 (46.3)	17 (40.5)	21 (52.2)	0.28
Numbers of metastatic oragns				0.32
1 site	13 (15.9)	5 (11.9)	8 (20.0)	
≥2 sites	69 (84.1)	37 (88.1)	32 (80.0)	
Prior regimens				
5‐fluorouracil	82 (100)	42 (100)	40 (100)	‐
Oxaliplatin	80 (97.6)	41 (97.6)	39 (97.5)	1^a^
Irinotecan	79 (96.3)	41 (97.6)	38 (95.0)	0.61^a^
Bevacizumab	70 (85.4)	35 (83.3)	35 (87.5)	0.59
Cetuximab	20 (24.4)	10 (23.8)	10 (25.0)	0.90
Regorafenib	4 (4.9)	1 (2.4)	3 (7.5)	0.35^a^
Fruquintinib	6 (7.3)	3 (7.1)	3 (7.5)	1^a^
Primary lesion excision				0.34
Yes	67 (81.7)	36 (85.7)	31 (77.5)	
No	15 (18.3)	6 (14.3)	9 (22.5)	
The time of received RT				
Before IT+TT (RT → IT + TT)		14 (33.3)		‐
After IT+TT (IT+TT → RT)		9 (21.4)		‐
Concurrent (RT − IT + TT)		19 (45.2)		‐
PD‐1 monoclonal antibody				
Sintilimab	64 (78.0)	30 (71.4)	34 (85.0)	‐
Nivolumab	3 (3.7)	1 (2.4)	2 (5.0)	‐
Camrelizumab	4 (4.9)	4 (9.5)	0 (0.0)	‐
Pembrolizumab	5 (6.1)	5 (11.9)	0 (0.0)	‐
Tislelizumab	2 (2.4)	1 (2.4)	1 (2.5)	‐
Toripalimab	4 (4.9)	1 (2.4)	3 (7.5)	‐
Targeting drugs				
Fruquintinib	68 (82.9)	33 (78.6)	35 (87.5)	0.28^a^
Regorafenib	14 (17.1)	9 (21.4)	5 (12.5)	
Gene mutation status^b^				
RAS wild	29 (43.3)	16 (48.5)	13 (38.2)	0.40
RAS mutant	38 (56.7)	17 (51.5)	21 (61.8)	
Lines of previous systemic therapy				
2 line	54 (65.9)	25 (59.5)	29 (72.5)	0.22
≥3 lines	28 (34.1)	17 (40.5)	11 (27.5)	
Cycles of TT + IT, median(range)	4 (2–26)	5 (2–20)	4 (2–26)	0.70

Abbreviations: ECOG PS, Eastern Cooperative Oncology Group performance status; IT, immunotherapy; PD‐1, programmed cell death 1; RT, radiotherapy; TT, targeted therapy.

^a^ Fisher's exact test.

^b^ Some data missing.

**TABLE 2:** Details of radiotherapy.

RT parameters	All (*n* = 42)
Purpose of RT, *n* (%)	
Curative RT	5 (11.9)
Palliative RT	37 (88.1)
RT site, *n* (%)	
Primary lesions	11 (26.2)
Liver	4 (9.5)
Lung	9 (21.4)
Bone	6 (14.3)
Abdominal wall	4 (9.5)
Retroperitoneal lymph nodes	4 (9.5)
Other sites*	4 (9.5)
Total prescribed dose (Gy)	
Median (range)	41 (10–56)
RT fraction	
Median (range)	10 (3–28)
Single RT dose, *n* (%)	
≥3Gy	27 (64.3)
≥5Gy	19 (45.2)

Abbreviation: RT, radiotherapy.

* Abdominal cavity and psoas major.

**TABLE 3:** Overall response.

Best response	Total, *n* (%)	RT (*n* = 42)	NRT (*n* = 40)	*p*‐value
CR	1 (1.2)	1 (2.4)	0 (0.0)	1
PR	10 (12.2)	8 (19.0)	2 (5.0)	0.11
SD	50 (61.0)	26 (61.9)	24 (60.0)	0.86
PD	21 (25.6)	7 (16.7)	14 (35.0)	0.06
ORR	11 (13.4)	9 (21.4)	2 (5.0)	0.03
DCR	61 (74.4)	35 (83.3)	26 (65.0)	0.06

Abbreviations: CR, complete response; DCR, disease control rate; ORR, overall response rate; PD, progressive disease; PR, partial response; SD, stable disease.

**TABLE 4:** Univariate and Multivariable Cox analysis of progression free survival and overall survival.

	PFS (*n* = 82)				OS (*n* = 82)			
	Univariate analysis HR (95%CI)	*p*‐value	Multivariate analysis HR (95%CI)	*p*‐value	Univariate analysis HR (95%CI)	*p*‐value	Multivariate analysis HR (95%CI)	*p*‐value
Age								
>56 vs ≤56	1.01 (0.63–1.62)	0.96			1.17 (0.69–1.97)	0.57		
Sex								
Female vs. male	1.02 (0.64–1.63)	0.94			0.95 (0.56–1.60)	0.83		
ECOG PS								
1 vs. 0	2.57 (1.52–4.32)	< 0.001	1.50 (0.84–2.69)	0.17	2.97 (1.60–5.51)	0.001	1.87 (0.93–3.73)	0.08
Cancer type								
Recurrent metastatic disease vs. primary metastatic disease	0.90 (0.56–1.44)	0.66			0.69 (0.40–1.18)	0.18		
Primary tumor location								
Left colon vs. rectum	0.66 (0.36–1.19)	0.17			0.65 (0.32–1.32)	0.23	0.83 (0.38–1.80)	0.63
Right colon vs. rectum	1.70 (0.97–2.97)	0.06			2.08 (1.12–3.87)	0.02	1.86 (0.96–3.64)	0.07
Primary lesion excision								
Yes vs. No	0.57 (0.31–1.06)	0.08			0.67 (0.35–1.28)	0.22		
Numbers of metastatic organs								
≥2 vs. 1	3.01 (1.36–6.68)	0.01	2.99 (1.30–6.90)	0.01	3.66 (1.31–10.22)	0.01	2.64 (0.80–8.77)	0.11
Liver metastases								
Yes vs. no	0.76 (0.47–1.24)	0.27			0.95 (0.56–1.64)	0.86		
Gene mutation status								
RAS wild vs. unknown	1.58 (0.78–3.23)	0.21			1.05 (0.49–2.24)	0.90		
RAS mutant vs. unknown	1.40 (0.70–2.77)	0.34			1.21 (0.54–2.70)	0.64		
Radiotherapy								
Yes vs. no	1.71 (1.06–2.76)	0.03	1.72 (1.03–2.87)	0.04	2.10 (1.22–3.60)	0.01	1.94 (1.05–3.59)	0.03
PD‐1 monoclonal antibody								
Sintilimab vs. other	0.78 (0.43–1.42)	0.42			0.66 (0.33–1.31)	0.24		
Targeting drugs								
Fruquintinib vs. other	0.79 (0.43–1.48)	0.47			0.90 (0.44–1.86)	0.78		
Lines of previous systemic therapy								
≥3 vs. 2	1.27 (0.78–2.09)	0.34			1.04 (0.60–1.82)	0.89		
Cycles of TT + IT								
>4 vs. ≤4	0.16 (0.09–0.30)	<0.001	0.19 (0.10–0.35)	<0.001	0.19 (0.10–0.36)	< 0.001	0.27 (0.13–0.56)	<0.001

Abbreviations: ECOG PS, Eastern Cooperative Oncology Group performance status; IT, immunotherapy; OS, overall survival; PD‐1, programmed cell death 1; PFS, progression‐free survival; RT, radiotherapy; TT, targeted therapy.

A total of seven patients with recurrent metastatic CRC were classified as were classified as synchronous metastasis. Given the inconsistency between metastatic and RT sites and the limited sample size, it was not analyzed in depth. The primary tumors were located in the rectum (*n* = 32, 39.0%), left colon (*n* = 24, 29.3%), and right colon (*n* = 26, 31.7%). Synchronous or metachronous liver metastases were present in 61.0% of CRC patients. Overall, 54 patients (65.9%) had previously received second‐line treatment, and 28 patients (34.1%) had received at least third‐line treatment. The majority of patients were treated with sintilimab and fruquintinib. The median treatment period for TT + IT is 4 cycles (range, 2–26). At the threshold of 1 month, RT sequential TT + IT was performed in 17 cases (40.5%), RT simultaneous TT + IT in 16 cases (38.1%), and TT + IT sequential RT in nine cases (21.4%). All patients were MSS/pMMR.

### 3.2 Efficacy

**FIGURE 2:**
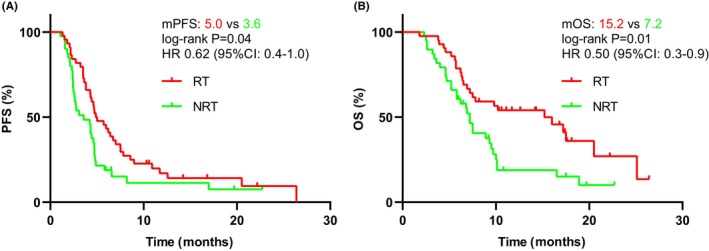
Kaplan–Meier survival curves of progression‐free survival (PFS) (A) and overall survival (OS) (B) according to anti‐angiogenesis targeted therapy combined with immunotherapy with (*n* = 42) or without RT (*n* = 40).

**FIGURE 3:**
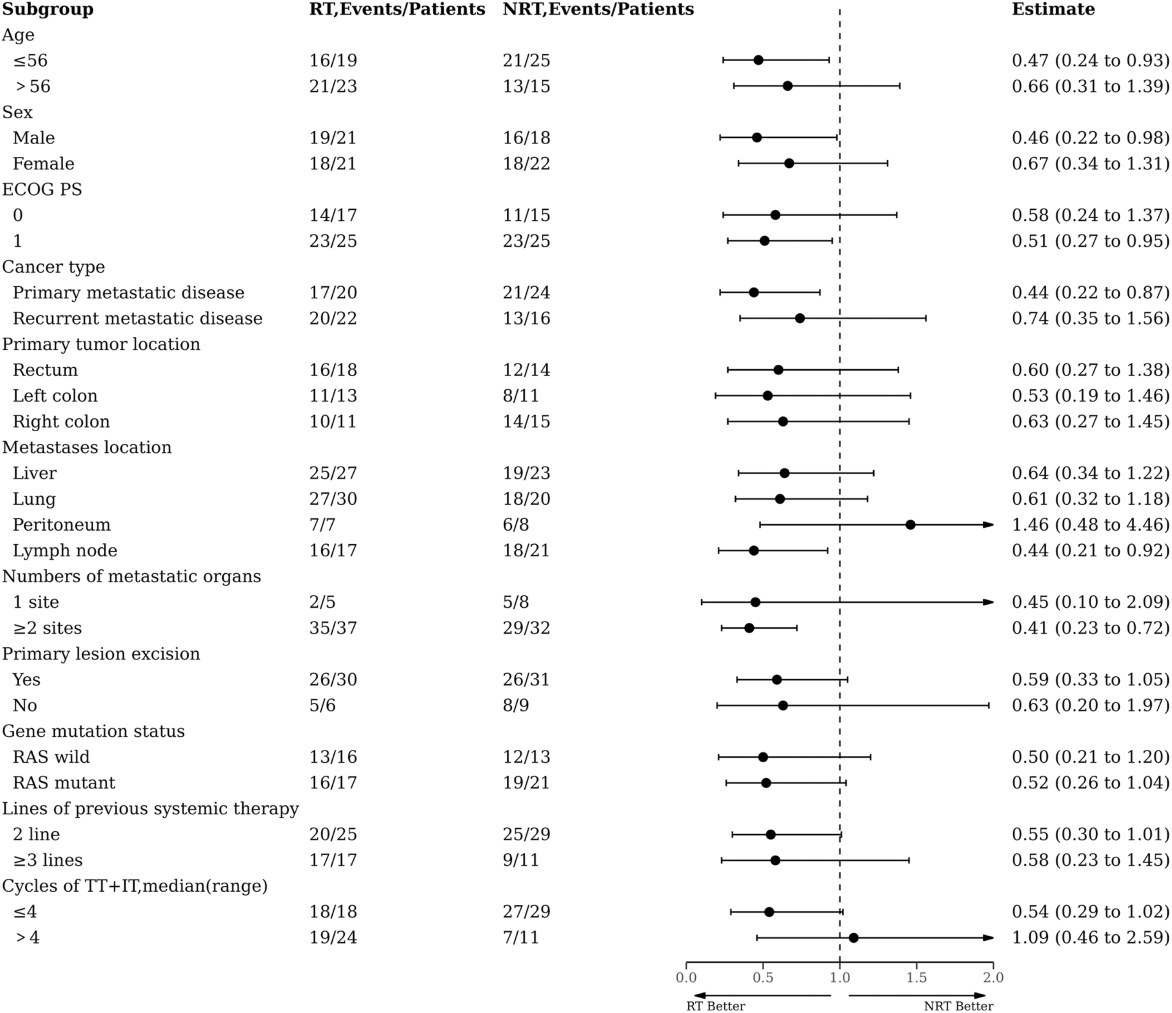
Forest plot of subgroup analysis on progression‐free survival (PFS).

The follow‐up cut‐off date was April 28, 2023, with a median follow‐up time of 17.5 months (95%CI: 14.7–20.3, 17.4 months in the RT group and 17.5 months in the NRT group). The mPFS was longer in the RT group than in the NRT group (5.0 months [95% CI: 3.4–6.6] vs. 3.6 months [95% CI: 2.0–5.1]; HR = 0.62 [95% CI: 0.4–1.0]; *p* = 0.04; Figure 2A). PFS subgroup analyses showed a greater preference for RT in all analyzed subgroups except for peritoneum metastases and the number of cycles of targeted combination IT (Figure 3). In addition, in the RT population, the mPFS of RT sequential/simultaneous TT + IT was superior to TT + IT sequential RT (RT → IT+TT vs. RT‐IT+TT vs. IT+TT → RT: 7.1 months vs. 6.2 months vs. 3.5 months, *p* = 0.004; Figure S1A). However, The PFS of patients in the TT + IT → RT group was shorter and comparable to that in the NRT group (3.5 months vs. 3.3 months, *p* = 0.599; Figure S2A).

**FIGURE 4:**
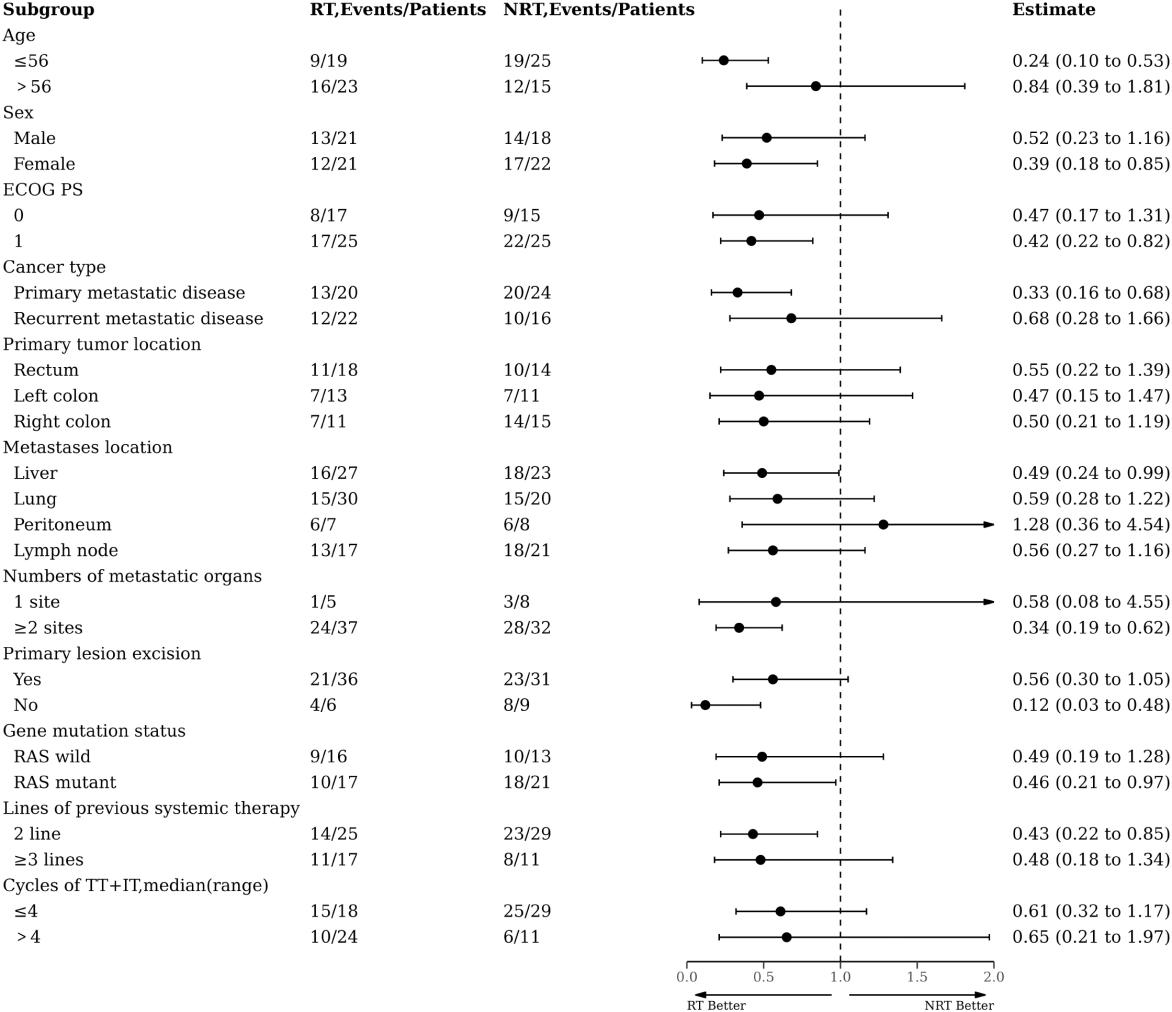
Forest plot of subgroup analysis on overall survival (OS).

The mOS was significantly longer in the RT group than in the NRT group (15.2 months [95% CI: 7.5–22.9] vs. 7.2 months [95% CI: 6.4–8.0]; HR = 0.50 [95% CI: 0.3–0.9]; *p* = 0.01; Figure 2B). Subgroup analysis of OS showed that RT was clinically beneficial for all subgroups analyzed, except for peritoneum metastases (Figure 4). Besides, it was observed that RT sequential/simultaneous TT + IT had an OS advantage over TT + IT sequential RT (14.2 months and 15.2 vs. 9.8 months; *p* = 0.40; Figure S1B). Nevertheless, we found no difference in OS between the TT + IT → RT group and the NRT group (9.8 months vs. 7.2 months, *p* = 0.426; Figure S2B).

We attempted to analyze the effect of RT on survival by single irradiation dose and irradiation site, taking into account the characteristics of the RT population in this study and the definition of single irradiation dose in the previous literature.^8^, ^9^ However, neither single doses <3Gy or ≥3Gy, nor single doses <5Gy or ≥5Gy, and primary or metastatic RT showed differences in PFS and OS (Figure S3A–F).

The treatment effect was summarized in Table 3. Among all populations, only one patient in the RT group achieved CR. The ORR was significantly higher in the RT group than in the NRT group (21.4% vs. 5.0%, *p* = 0.03), and the DCR showed the same trend, albeit without achieving statistical significance (83.3% vs. 65.0%, *p* = 0.06).

To further determine the influencing factors of PFS and OS, univariate analysis was carried out, and then those with *p* < 0.05 were included in multivariate analysis (Table 4). In a univariate analysis, ECOG PS = 0, the number of metastases involving less than two organs, undergoing RT, and more than four cycles of TT + IT were connected with a better PFS. The ECOG PS was no longer a prognostic factor for PFS in the multivariate analysis. And in univariate analysis, five factors were associated with better OS. However, in multivariate analysis, only RT and more than four cycles of TT + IT were an independent prognostic indicator with significantly better OS (HR = 1.94, 95%CI: 1.05–3.59, *p* = 0.03; HR = 0.27, 95%CI: 0.13–0.56, *p* < 0.001).

### 3.3 Safety

TRAEs were mainly manifested as anti‐angiogenic targeted drug‐related adverse effects (such as bleeding, hypertension, and hand‐foot syndrome) and IT‐related adverse reactions (such as pneumonia, myocarditis, and hypothyroidism). The overall tolerance of the patients in the RT group was good, and one patient developed radiation pneumonia. No treatment‐related death was reported. All TRAEs were manageable with active treatment.

## 4 DISCUSSION

The effectiveness of anti‐angiogenic targeted drugs combined with ICIs in the treatment of pMMR/MSS mCRC at the third line or above has been confirmed in several prospective clinical trials (Table S1). However, whether the implementation of RT can further improve the survival benefit in this subset of CRC patients is not yet known. Fortunately, our results showed that RT combined with anti‐angiogenic targeted therapy (TT) and ICIs could prolong PFS and OS in patients with pMMR/MSS mCRC without accidental toxicity. Compared with the NRT group, the RT group had a mPFS of 5.0 months and a 38% lower risk of disease progression or death. It is particularly encouraging that the OS of the RT group is significantly better than that of the NRT group (15.2 m vs. 7.2 m), with a 50% reduction in the risk of death.

Notably, subgroup analyses of both PFS and OS concluded that the combination RT modality was more beneficial to pMMR/MSS mCRC. Moreover, in line with previous studies reporting that RT sequential ICIs or synchronous ICIs was more helpful to activate anti‐tumor effects, the results of the present study showed that the use of RT prior to targeted drugs and PD‐1 inhibitors combination or synchronous application was superior to the use of RT after targeted drugs and PD‐1 inhibitors combination in terms of both PFS and OS. The reasons may be as follows: RT can induce immunogenic death of tumor cells, release new tumor‐associated antigens, promote the antigen presentation function of dendritic cells, and increase the infiltration of effector T lymphocytes.^10^ Anti‐angiogenic drugs combined with ICIs can reshape the tumor vascular microenvironment and immune microenvironment and play a synergistic anti‐tumor effect.^11^ Therefore, RT can enhance the effect of sequential TT + IT. However, induction IT can change the tumor microenvironment, promote tumor angiogenesis and increase the distribution of oxygen, which has a synergistic effect with RT.^11^, ^12^ But at the same time, TT inhibits tumor angiogenesis, resulting in hypoxia leading to RT resistance.^13^ Therefore, this may be the reason for the relatively poor efficacy of the patients treated with sequential RT. In addition, we observed that the TT + IT sequential RT group did not show an advantage in PFS and OS compared to the NRT group, which further exemplified the survival advantage of RT sequential or synchronous TT + IT.

To fully interpret the benefits of RT, we attempted to analyze the effects of RT site and dose on PFS and OS. However, due to the limitation of sample size and the heterogeneity of the treatment regimen, it is impossible to accurately analyze a specific RT site or dose. Based on the analysis results in Figure S3, no influence of different RT sites and doses on treatment outcomes was found. In fact, the effect of RT site and dose on the degree of immune enhancement cannot be underestimated, which needs to be further explored in future studies.

In multivariate analysis, the introduction of RT and the number of cycles of TT and IT combinations were co‐independent influences on PFS and OS. The addition of RT was also associated with a higher ORR and DCR (21.4% vs. 5.0%, *p* = 0.03; 83.3% vs. 65.0, *p* = 0.06), and no inferior overall safety compared to anti‐angiogenic therapy combined with ICIs. The most common AEs were still those related to targeted drugs and ICIs; RT did not increase the extent of toxic side effects and only one patient had a RT‐related AE (radiation pneumonitis), which was controllable after active intervention. In general, these findings may provide a valuable reference for implementing RT in patients with pMMR/MSS mCRC. Similarly, the 2022 Chinese Society of Clinical Oncology reported on a study of the efficacy and safety of multifocal stereotactic ablative radiotherapy (SABR) in combination with furoquinitinib and tislelizumab in the treatment of mCRC patients (NCT04948034), with preliminary results of 13 patients with an ORR of 23.1% and a DCR of 61.5%. And 11 patients survived and eight are still undergoing treatment.^14^ This preliminary result further confirms the improved efficacy of RT for CRC patients receiving targeted combination IT. Despite the differences in the dose and pattern of RT between this retrospective study and that prospective study, the overall advantages of RT can still be highlighted.

This study also has limitations: (1) The study was a single‐center retrospective study with a limited sample size for inclusion. (2) The variety of antivascular targeting drugs and PD‐1 inhibitors, as well as the difference in RT sites and time, affect the consistency of the treatment process. (3) Detailed subgroup analysis of patients in the RT group could not be performed due to the limitation of the number of cases. In the future, it is necessary to select the best RT sites and determine the time of RT and fractionation dose to maximize survival benefits. (4) Flawed by retrospective studies, some treatment‐related toxicities may have been underestimated. Thus, it is necessary to design prospective studies to determine the efficacy and safety of the combination of RT, PD‐1/PD‐L1 inhibitors, and anti‐angiogenic drugs.

## 5 CONCLUSIONS

The addition of RT significantly improves the survival benefit of anti‐angiogenic drugs combined with ICIs for MSS/pMMR mCRC patients, with a favorable safety profile. Further, RT sequential or simultaneous antiangiogenic TT combined with ICIs may be the optimal combination sequence. However, due to the heterogeneity of the treatment regimens involved in this retrospective study, neither the RT group nor the NRT group fully belongs to the scope of standard treatment, and there is no conclusion on how to compare with standard care. Nonetheless, using the available data and strictly balancing the differences between the groups, we still demonstrated the survival benefit advantage of RT in TT combined with IT that warrants further exploration in the future.

## AUTHOR CONTRIBUTIONS

**Menglan Zhai:** Conceptualization (equal); data curation (equal); formal analysis (equal); writing – original draft (equal); writing – review and editing (equal). **Zixuan Zhang:** Data curation (equal); formal analysis (equal); writing – original draft (equal). **Haihong Wang:** Data curation (equal); investigation (equal); software (equal); supervision (equal). **Jinghua Ren:** Data curation (equal); formal analysis (equal); supervision (equal); validation (equal). **Sheng Zhang:** Project administration (equal); supervision (equal); validation (equal). **Mingjie Li:** Data curation (equal); formal analysis (equal). **Lichao Liu:** Data curation (equal); formal analysis (equal). **lisha li:** Formal analysis (equal); software (equal). **Lan Zhang:** Formal analysis (equal); supervision (equal). **Xin Li:** Supervision (equal); validation (equal). **Tao Zhang:** Funding acquisition (equal); supervision (equal); validation (equal); visualization (equal). **Zhenyu Lin:** Conceptualization (equal); formal analysis (equal); funding acquisition (equal); writing – review and editing (equal).

## FUNDING INFORMATION

This work was supported by Wuhan Union Hospital Innovation Research (2021xhyn063), Shanghai Cancer Prevention and Anti‐Cancer Development Foundation 2020 Discovery research funding (2020HX025), Chinese Society of Clinical Oncology (CSCO)‐Tongshu Oncology Research Fund (Y‐tongshu2021/qn‐0205), and CSCO‐Xinda Oncology Immunotherapy Research Fund (Y‐XD202002‐0168).

## CONFLICT OF INTEREST STATEMENT

All authors declare to have no conflict of interest.

## ETHICS STATEMENT

Study has been approved by the Institutional Review Board of Union Hospital, Tongji Medical College, Huazhong University of Science and Technology. The requirement for informed consent was waived owing to the retrospective nature of the study.

## Supporting information


Figure S1.

Figure S2.

Figure S3.

Table S1.

## Data Availability

The datasets generated during and/or analyzed during the current study are available from the corresponding author on reasonable request.
