# Supplementary figures and images for: Efficacy and safety of radiotherapy combined with anti‐angiogenic therapy and immune checkpoint inhibitors in MSS/pMMR metastatic colorectal cancer
Source: Cancer Med. 2023 Dec 19;13(1):e6820. doi: 10.1002/cam4.6820 (PMC10807612; doi:10.1002/cam4.6820)

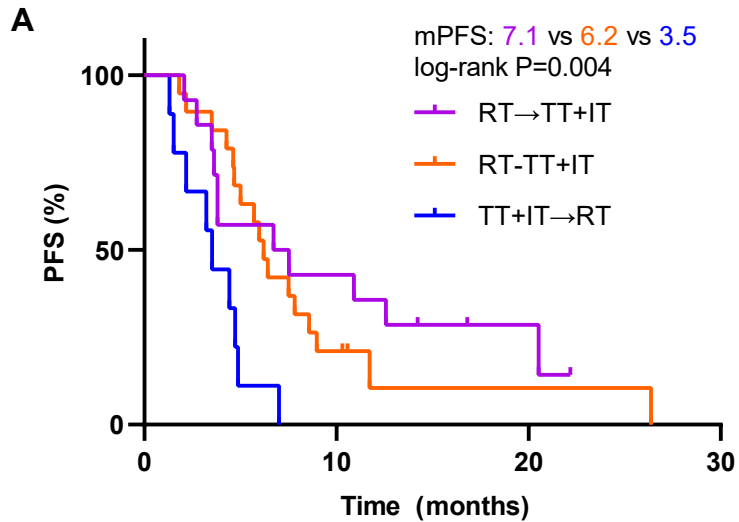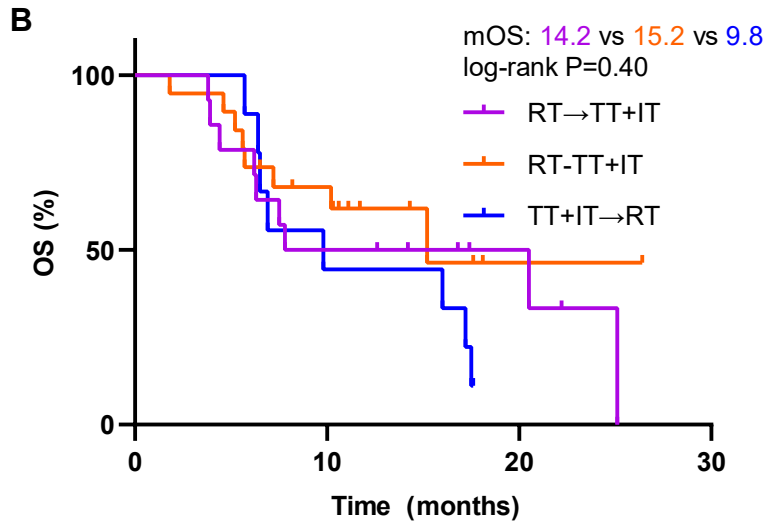

Supplement: Supplementary file 1 — Figure S1. [file CAM4-13-e6820-s003.pdf]

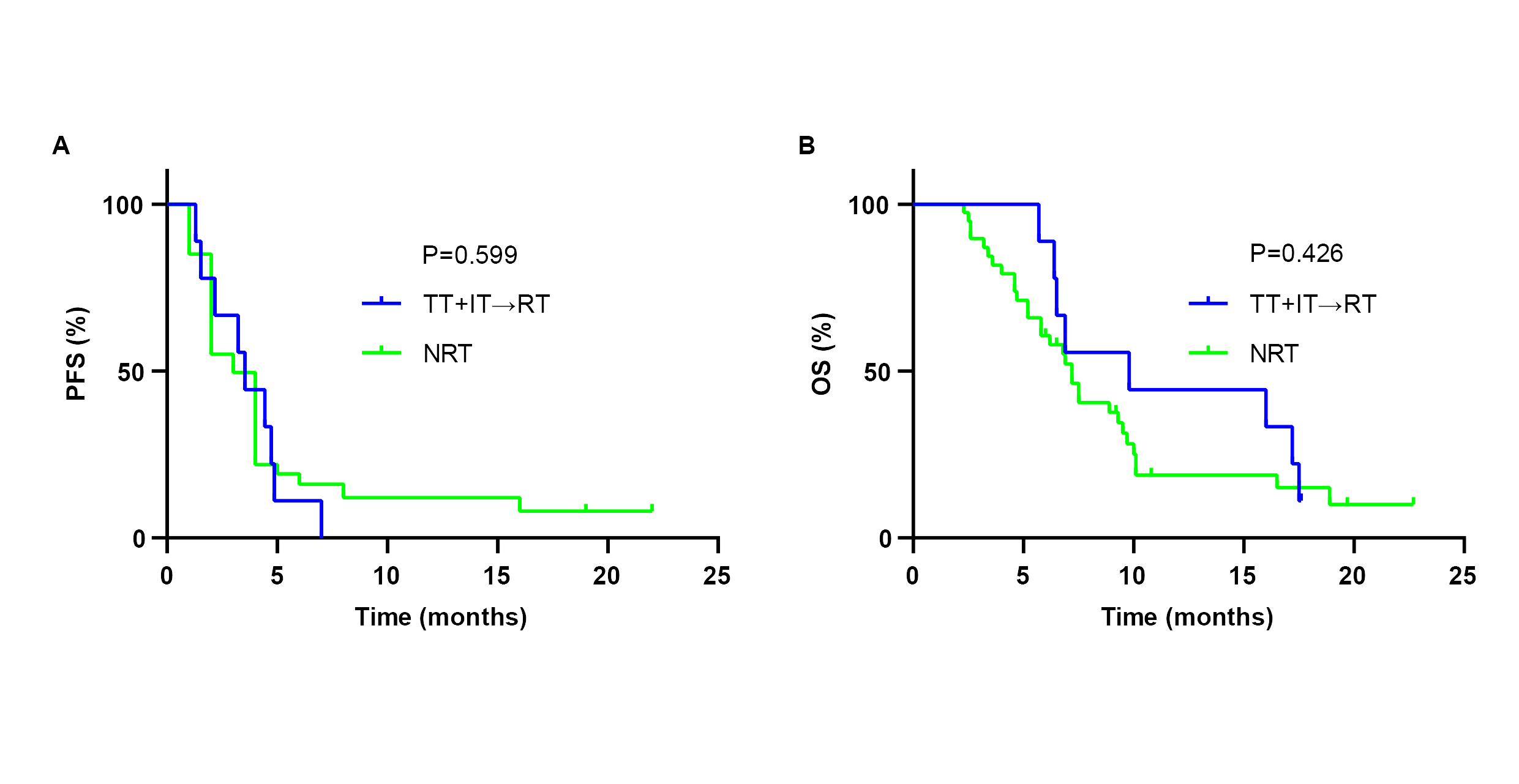

Supplement: Supplementary file 2 — Figure S2. [file CAM4-13-e6820-s002.tif]

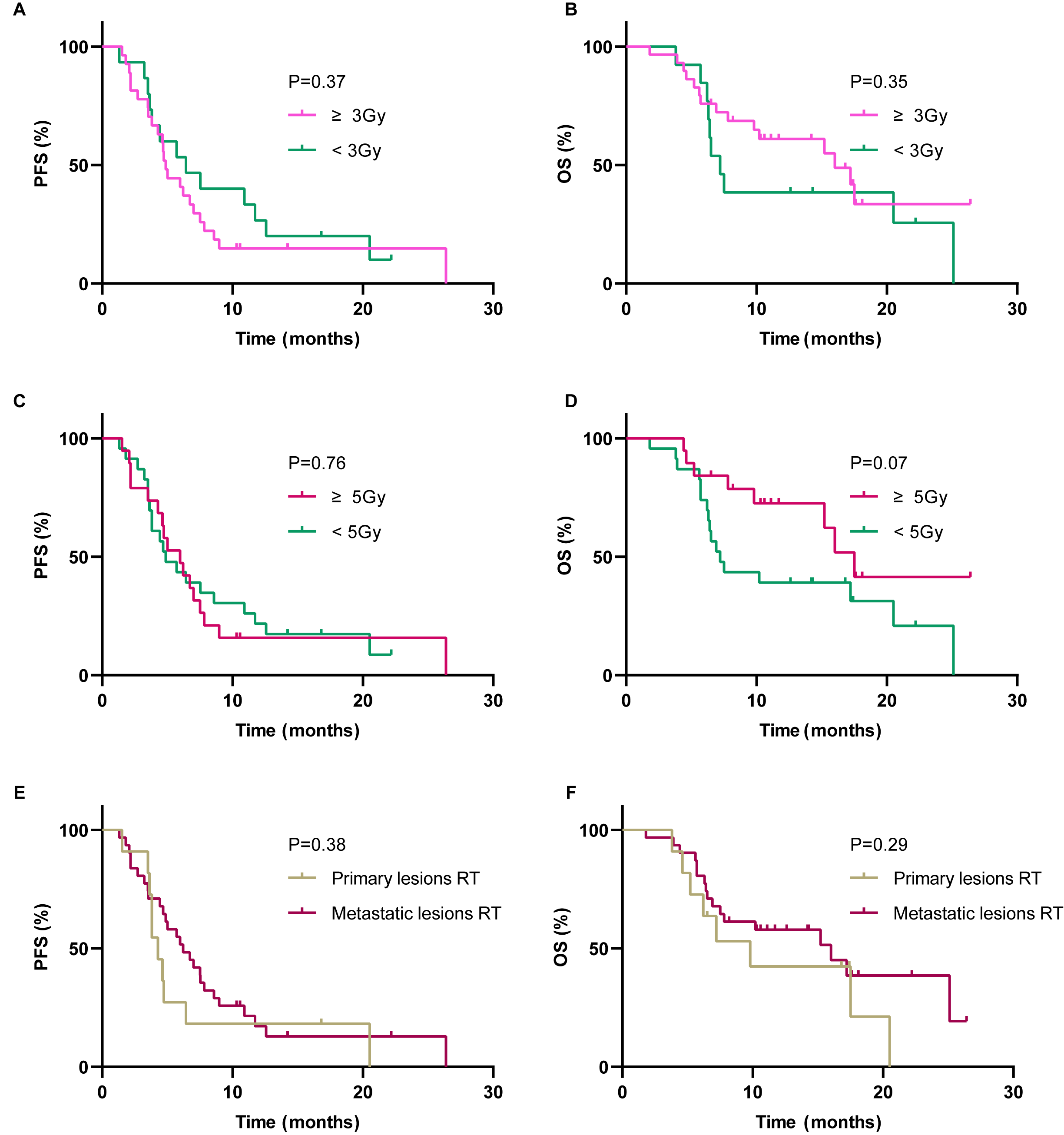

Supplement: Supplementary file 3 — Figure S3. [file CAM4-13-e6820-s004.tif]
