# Supplementary material for: Efficacy and safety of radiotherapy combined with anti‐angiogenic therapy and immune checkpoint inhibitors in MSS/pMMR metastatic colorectal cancer
Source: Cancer Med. 2023 Dec 19;13(1):e6820. doi: 10.1002/cam4.6820 (PMC10807612; doi:10.1002/cam4.6820)
Supplement: Supplementary file 4 — Table S1. [file CAM4-13-e6820-s001.docx]

**Table S1** A summary of studies on the treatment of mCRC with antiangiogenic drugs combined with immune checkpoint inhibitors (reported)

| **Study** | **Published time** | **MSI/MMR state** | **Treatment lines** | **Regimen** | **Cases** | **ORR** | **DCR** | **mPFS (m)** | **mOS (m)** |
| --- | --- | --- | --- | --- | --- | --- | --- | --- | --- |
| REGONIVO^1^ | 2019-ASC0/2020ASC0-GI | MSS 96%, MSI-H 4% | Standard treatment failure | Regorafenib 80/120/160mg, d1-21, Q4W; Nivolumab 3mg/kg, d1, Q2W | 25 | 33% (MSS) | - | 7.9 | not reached |
| NCT03903705^2^ | 2020-ASCO | non-MSI-H/pMMR | Standard treatment failure | Fruquintinib 3mg, 3W/1W; Sintilimab 200mg d1, Q3W | 52 | 15.38% | 57.69% | 3.6 | - |
| REGOMUNE^3^ | 2020-ASCO | non-MSI-H | third line | Regorafenib 160mg, Qd, 3W/1W；Avelumab 10mg/kg, d1, Q2W | 48 | 0 (30% had reduction in tumor burden) | 57.50% | 3.6 | 10.8 |
| REGOTORI^4^ | 2020-EMSO | MSS/pMMR/MSI-L | Third line and above | Regorafenib 80mg, d1-21, Q4W; Toripalimab 3mg/kg, d1, d15, Q4W | 33 | 15.20% | 36.40% | 2.1 | 15.5 |
| NCT04126733^5^ | 2021-ASCO | MSS/pMMR | Standard treatment failure | Regorafenib 80-120, d1-21, Q4W；Nivolumab 480mg, d1, Q4W | 70 | 7% (Liver metastases 0%) | 39% | 1.8 | 11.9 |
| LEAP005^6^ | 2021-ASCO | non-MSI-H/pMMR | Second line and above | Lenvatinib 20mg, Qd; Pembrolizumab 200mg, d1, Q3W | 32 | 22% | 47% | 2.3 | 7.5 |
| NCT03903705^7^ | 2022 | MSS | Third line and above | Fruquintinib 5mg, Qd, 2W/1W or 3mg, Qd; Sintilimab 200mg, Q3W | 25 (5mg: 15, 3mg: 10) | 20% (5mg：20%, 3mg：20%) | 92% (5mg: 100%, 3mg: 80%) | 6.9 (5mg: 6.9, 3mg：5.6) | 17.3 (5mg: 20, 3mg: 13.9) |

**Table S1** Continue

| **Study** | **Published time** | **MSI/MMR state** | **Treatment lines** | **Regimen** | **Cases** | **ORR** | **DCR** | **mPFS (m)** | **mOS (m)** |
| --- | --- | --- | --- | --- | --- | --- | --- | --- | --- |
| NCT03977090^8^ | 2021-ASCO | MSS 80%, MSI-H 6.7%, 13.3% unknown | Second line and above | Fruquintinib Qd (3/4/5mg), 3W/1W; Geptanolimab 3mg/kg, d1, Q2W | 15 | 26.7% (MSS 25%) | 80% (MSS 75%) | 7.3 (MSS:5.45) | not reached |
| RIN^9^ | 2022-EMSO | MSS | Standard treatment failure | Regorafenib 80mg,d1-21,Q4W; Ipilimumab1mg/kg, Q6W and Nivolumab 240mg, Q2W | 29 | 27.60% | 62.10% | 14 | -- |
| NCT04695470^10^ | 2022-EMSO | MSS/pMMR | Third line and above | Fruquintinib 5mg, d1-14, Q3W; Sintilimab 300mg, d1, Q3W | 43 | 16% | 77% | 4.1 | estimate 13.3 |

MMR/MSI: mismatch repair/microsatellite instability; MSI-H: high microsatellite instability; MSS-L: low microsatellite instability; MSS: microsatellite stable; pMMR: mismatch repair profificient; DCR: disease control rate; ORR: objective response rate.

**REFERENCES**

1. Fukuoka S, Hara H, Takahashi N, Kojima T, Kawazoe A, Asayama M, et al. Regorafenib Plus Nivolumab in Patients With Advanced Gastric or Colorectal Cancer: An Open-Label, Dose-Escalation, and Dose-Expansion Phase Ib Trial (REGONIVO, EPOC1603). *J Clin Oncol.* 2020;38(18):2053-2061. https://doi.org/10.1200/JCO.19.03296
2. Gou M, Yan H, E Liu, Wang Z, Si H, Chen Set al. Fruquintinib combination with sintilimab in refractory metastatic colorectal cancer patients in China. *J Clin Oncol.* 2020;38(15_suppl):4028-4028. https://doi.org/10.1200/JCO.2020.38.15_suppl.4028
3. Sophie Cousin CAB, Jean Philippe Guégan CAG, Jean-Philippe Metges AA, Carlos A. Gomez-Roca, Jean-Philippe Metges, Antoine Adeniset al. REGOMUNE: A phase II study of regorafenib plus avelumab in solid tumors—Results of the non-MSI-H metastatic colorectal cancer (mCRC) cohort. *J Clin Oncol.* 2020;38:15_suppl, 4019-4019. https://doi.org/[10.1200/JCO.2020.38.15_suppl.4019](http://dx.doi.org/10.1200/JCO.2020.38.15_suppl.4019)
4. Wang F, He MM, Yao YC, Zhao X, Wang ZQ, Jin Y, et al. Regorafenib plus toripalimab in patients with metastatic colorectal cancer: a phase Ib/II clinical trial and gut microbiome analysis. *Cell Rep Med.* 2021;27;2(9):100383. https://doi.org/10.1016/j.xcrm.2021.100383
5. Fakih M, Raghav KP, Chang DZ, [Bendell](https://www.semanticscholar.org/author/J.-Bendell/5877371) JC, [Larson](https://www.semanticscholar.org/author/T.-Larson/2070370255) T, [Cohn](https://www.semanticscholar.org/author/A.-Cohn/2600564) AL, et al. Single-arm, phase 2 study of regorafenib plus nivolumab in patients with mismatch repair⁃proficient (pMMR) / microsatellite stable (MSS) colorectal cancer (CRC). *J Clin Oncol.* 2021;39(Suppl 15):abstr3560. https://doi.org/10.1200/JCO.2021.39.15_suppl.3560
6. Carlos GR, Eduardo Y, Seock-Ah I, Eduardo A, Helene S, Mark D, et al. LEAP-005: A phase II multicohort study of lenvatinib plus pembrolizumab in patients with previously treated selected solid tumors-Results from the colorectal cancer cohort. *J Clin Oncol.* 2021;39(3_suppl):94-94. https://doi.org/10.1200/JCO.2021.39.3_suppl.94
7. Guo Y, Zhang W, Ying J, Zhang Y, Pan Y, Qiu W, et al. Phase 1b/2 trial of fruquintinib plus sintilimab in treating advanced solid tumours: The dose-escalation and metastatic colorectal cancer cohort in the dose-expansion phases. *Eur J Cancer.* 2023;181:26-37. https://doi.org/10.1016/j.ejca.2022.12.004
8. Bai Y, Xu N, An S, Chen W, Gao C, ZhangD. A phase ib trial of assessing the safety and preliminary efficacy of a combination therapy of geptanolimab (GB 226) plus fruquintinib in patients with metastatic colorectal cancer(mCRC). *J Clin Oncol.* 2021;39,no. 15_suppl. https://doi.org/10.1200/JCO.2021.39.15_suppl.e15551
9. Fakih MG, Sandhu1 J, Lim D, Li SM, Wang C. A phase I clinical trial of regorafenib, ipilimumab, and nivolumab (RIN) in chemotherapy resistant MSS metastatic colorectal cancer (mCRC). *Ann Oncol.* 2022;33 (suppl_7): S136-S196. https://doi.org/10.1016/annonc/annonc1048
10. Zhang W, Sun Y, Jiang Z, Liu T, Gong C, Yang L, etal. Fruquintinib plus sintilimab in refractory repair-proficient (pMMR)/microsatellite stable (MSS) metastatic colorectal cancer (mCRC): Preliminary clinical results and biomarker analyses from a phase II study. *Ann Oncol.* 2022;33 (suppl_7):S136-S196. <https://doi.org/10.1016/annonc/annonc10>
